# Acyclovir Nephrotoxicity: A Case Report Highlighting the Importance of Prevention, Detection, and Treatment of Acyclovir-Induced Nephropathy

**DOI:** 10.1155/2010/602783

**Published:** 2010-08-31

**Authors:** Raymond Fleischer, Michael Johnson

**Affiliations:** ^1^Internal Medicine, University of Texas Health Science Center, San Antonio, TX 78229, USA; ^2^Division of Hospital Medicine, University of Texas Health Science Center, San Antonio, TX 78229, USA

## Abstract

Acute kidney injury is an unfortunate complication of acyclovir therapy secondary to crystal-induced nephropathy. It is characterized by a decrease in renal function that develops within 24–48 hours of acyclovir administration indicated by a rapid rise in the serum creatinine. Failure to quickly realize this as an etiology of acute kidney injury can lead to excessive morbidity to the patient. The case described in this vignette is an example of the clinical manifestation of acyclovir crystal obstructive nephrotoxicity. We will briefly discuss the pathophysiology, diagnosis, prevention, and management of patients that present with acyclovir nephrotoxicity.

## 1. Introduction

Acute kidney injury is a well-described side effect of acyclovir, the most common mechanism being crystal nephropathy. Unfortunately, although the side-effects of acyclovir are well known, they are often underappreciated. Acute kidney injury secondary to acyclovir is characterized by a decrease in renal function that usually develops within 12–48 hours of drug administration as indicated by a rapid rise in the serum creatinine (S-Cr) [1]. Immediate detection of acute kidney injury is necessary to prevent morbidity [2]. This paper will review the clinical presentation of acyclovir (Zovirax) nephrotoxicity and discuss potential mechanisms for its prevention and treatment.

## 2. Case Description

### 2.1. History

Mr. A, a 25-year-old male with a past medical history of bipolar disorder, was seen in an outpatient clinic 3 days prior to admission where he was started on oral acyclovir 800 mg 5 times a day and vicodin for a diagnosis of Herpes Zoster. He then presented to the emergency department with a one-day history of pruritis, redness, and blurry vision of the left eye. In addition, he stated the vesicular rash that was covering a portion of his right lower abdominal quadrant and right flank was worsening. The rash was accompanied by a severe burning pain. Outpatient medications included abilify, xanax, ambien, propranolol, and trileptal. 

### 2.2. Physical Exam

Afebrile. Oropharynx: moist. Neck: supple. Heart: regular without murmurs. Lungs: clear to auscultation bilaterally. Thorax: unremarkable on admission but developed mild costovertebral tenderness on Day 3. Extremities: no edema. Skin: multiple crops of vesicles in different stages on the right lower abdominal quadrant and right flank consistent with T12 and L1 dermatomes. Initial exam by ophthalmologist was concerning for a corneal ulceration but subsequent examination revealed only corneal edema.

### 2.3. Studies

Admission S-Cr 0.9, Peak S-Cr 5.4 with BUN 31 (Normal Ranges for S-Cr and BUN are 0.6–1.2 mg/dL and 7–18 mg/dL). Urinalysis collected after rise in S-Cr was negative for RBCs, WBCs, granular casts, and eosinophils; it was also negative for crystals although polarizing light microscopy was unavailable to adequately look for crystals. Renal sonogram after S-Cr elevation was normal. HIV negative.

### 2.4. Hospital Course

**Figure 1:**
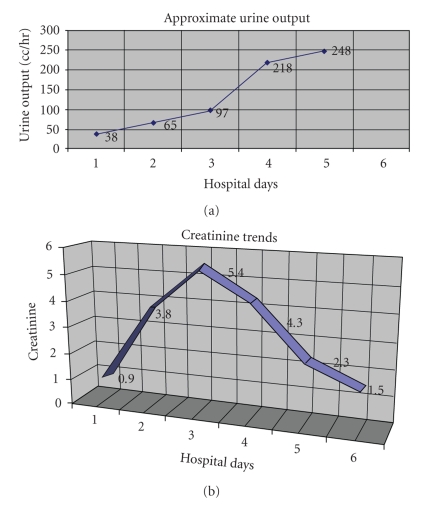
One dose of 800 mg of IV acyclovir was given on Day 1 and then the patient received one dose of 800 mg orally that night. On the morning of Day 2, the acute kidney injury was found and acyclovir was stopped immediately. The patient had received 800 mg of acyclovir orally five times a day for three days before hospital admission (prior to Day 1).

The patient was admitted to the general medicine wards but was started on IV acyclovir for herpes ophthalmicus by the ophthalmology team while still in the emergency department. He had not been on IV fluids when he received the IV dose of acyclovir. Subsequent workup was negative for herpes ophthalmicus and IV acyclovir was discontinued; unfortunately, by hospital Day 2, the patient's S-Cr had more than tripled since admission. Crystal-induced nephropathy was suspected and the patient was started on aggressive IV fluids. Although euvolemic, furosemide was added to maintain a urine output of at least 150 cc/hr. Further evaluation for other causes of acute kidney injury was unrevealing (see Section 2.3) including a negative history for exposure to other nephrotoxic agents (NSAIDS, etc.) prior to, or during, his hospitalization. After peaking on hospital Day 3, the patient's S-Cr began to decline back to baseline over the following three days. The total amount of acyclovir given was a one-time dose of 800 mg IV and a total of 13.6 grams by mouth prior to the development of acute kidney injury. The timing of the acute kidney injury correlated with the IV dose (see Figure 1 for graphical representation). 

## 3. Case Overview

The patient in this case rapidly developed acute kidney injury after receiving about 14 grams of acyclovir, including one IV dose approximately 24 hours before the serum creatinine began to climb. He had no other risk factors for developing acute kidney injury and was not volume depleted on examination. He was relatively asymptomatic but did complain of flank pain on days 3–5 of the hospital stay which has been reported in other cases of acyclovir induced nephropathy [1]. After maintaining his urine output at a high rate with IV fluids and furosemide, his renal function began to improve towards baseline.

## 4. Discussion

This case describes a patient with no underlying comorbidities developing acute kidney injury secondary to crystal nephropathy after receiving just one IV dose of acyclovir. Other potential mechanisms of injury include acute interstitial nephritis (AIN) and acute tubular necrosis (ATN); however, the most commonly described mechanism is obstructive nephropathy [3]. In this particular case, urinalysis and peripheral blood smear did not support an ATN or AIN diagnosis. The basis for a diagnosis of crystal-induced nephropathy in this patient is supported by the clinical history and time course of the acyclovir administration. Urinalysis did not show crystals, although without polarizing light microscopy, they would have been difficult to see. In addition, the patients kidney injury was noticed immediately and measures were taken to increase urinary flow rates before the urinalysis could be collected. With these two factors, it was not surprising to us that crystals were not seen. There has been some evidence in animal models that acute kidney injury can occur secondary to acyclovir administration without crystal obstruction, but from effects on renal microcirculation, although the exact mechanism is unknown [3]. Although there is a small chance of acute kidney injury without crystal obstruction, there has been sufficient evidence to show that kidney injury secondary to crystal obstruction is the most common and dominant mechanism and thus the likely diagnosis in this case [1]. Acyclovir, which is relatively insoluble in urine, is rapidly filtered by the glomeri and secreted by the renal tubules which can produce high urine concentrations, especially in patients with decreased urine flow rates [1]. Oral acyclovir has poor bioavailability and usually only causes nephropathy when the patient is severely volume depleted or at high doses in relation to renal function [1]. IV administration is necessary to achieve high blood concentrations, which explains why crystal nephropathy is more common with IV administration. Renal excretion accounts for 60%–90% of acyclovir elimination [1]. When intratubular deposition of crystals occurs, the nephron becomes obstructed leading to increased resistance to renal blood flow and subsequent elevation of the S-Cr [1, 3]. The importance of monitoring renal function in hospitalized patients on acyclovir is strongly supported by this case presentation. The possibility for chronic kidney injury is a strong concern if the renal insufficiency is not rapidly detected [2]. In addition, there is strong evidence that acyclovir/valacyclovir can also cause neurotoxicity in the setting of kidney injury, which could further complicate the patient's clinical picture [4]. Common laboratory findings other than an increase in S-Cr include hematuria and pyuria on urinalysis, as well as birefringent needle-shaped crystals seen on polarizing light microscopy [1]. The risk of acyclovir induced nephropathy can be minimized with empiric IV fluids to establish euvolemia before drug administration, avoiding rapid infusion of the drug (infuse slowly over 1-2 hours), and adjusting the dose for renal function if necessary [1]. Other nephrotoxic drugs such as aminoglycosides and cyclosporine also place the patient at a higher risk of developing kidney injury with acyclovir therapy. Treatment of acyclovir nephrotoxicity is supportive with discontinuation or reduction of the drug in addition to maintaining a high urinary flow rate (>150 cc/hr) with IV fluids and furosemide [1]. There is limited evidence that famciclovir may be substituted for acyclovir if renal toxicity develops for treatment of certain infections [5]. In patients that develop severe renal failure or do not respond to treatment, hemodialysis is an option to remove the offending drug and support renal function [1]. Acyclovir is commonly used in transplant patients, herpes virus, and varicella zoster virus infections, and other patients who present with potential viral CNS infections; hence, clinicians must be familiar with the potential side effects associated with its use and how drug-associated complications can be treated.

## Figures and Tables

**Table 1:** Risks, diagnosis, prevention, and treatment of acyclovir crystal nephropathy.

Risk factors	Laboratory and clinical findings	Prevention	Treatment
Hypovolemia	Increased Cr, rapid and usually within 12–48 hours	Establish euvolemia before medication administration	If possible, discontinue or reduce dose
Rapid IV infusion	Pyuria	Infuse drug slowly (over 1-2 hours)	Establish high urinary flow with IV fluids and furosemide (>150 cc/hr)
Concurrent acute kidney injury before medication administration	Hematuria	Adjust dose for renal function	Hemodialysis if necessary
Excess medication dosage in relation to renal function	Birefringent Needle-shaped crystals	Avoid other nephrotoxic agents	May replace acyclovir with famciclovir in certain instances while increasing urinary flow rate
Concurrent use of other nephrotoxic agents	Pt. may complain of associated flank pain		
	Pt. may be oliguric		

IV: intravenous, Cr: Creatinine, cc: milliliters, and hr: hour
